# Racism as the fundamental cause of ethnic inequities in COVID-19 vaccine hesitancy: A theoretical framework and empirical exploration using the UK Household Longitudinal Study

**DOI:** 10.1016/j.ssmph.2022.101150

**Published:** 2022-06-24

**Authors:** Laia Bécares, Richard J. Shaw, Srinivasa Vittal Katikireddi, Patricia Irizar, Sarah Amele, Dharmi Kapadia, James Nazroo, Harry Taylor

**Affiliations:** a Department of Social Work and Social Care, University of Sussex, Falmer, UK; b MRC/CSO Social and Public Health Sciences Unit, University of Glasgow, Glasgow, UK; c Department of Sociology, University of Manchester, Manchester, UK; d Department of Social Statistics, University of Manchester, Manchester, UK

**Keywords:** Racism, Ethnic inequities, Vaccine hesitancy, COVID-19

## Abstract

Ethnic inequities in COVID-19 vaccine hesitancy have been reported in the United Kingdom (UK), and elsewhere. Explanations have mainly focused on differences in the level of concern about side effects, and in lack of trust in the development and efficacy of vaccines. Here we propose that racism is the fundamental cause of ethnic inequities in vaccine hesitancy. We introduce a theoretical framework detailing the mechanisms by which racism at the structural, institutional, and interpersonal level leads to higher vaccine hesitancy among minoritised ethnic groups. We then use data from Wave 6 of the UK Household Longitudinal Study COVID-19 Survey (November to December 2020) to empirically examine these pathways, operationalised into institutional, community, and individual-level factors. We use the Karlson–Holm–Breen method to formally compare the relationship between ethnicity and vaccine hesitancy once age and gender, sociodemographic variables, and institutional, community, and individual-level factors are accounted for. Based on the Average Partial Effects we calculate the percentage of ethnic inequities explained by each set of factors.

Findings show that institutional-level factors (socioeconomic position, area-level deprivation, overcrowding) explained the largest part (42%) of the inequity in vaccine hesistancy for Pakistani or Bangladeshi people, and community-level factors (ethnic density, community cohesion, political efficacy, racism in the area) were the most important factors for Indian and Black groups, explaining 35% and 15% of the inequity, respectively.

Our findings suggest that if policy intervened on institutional and community-level factors – shaped by structural and institutional racism - considerable success in reducing ethnic inequities might be achieved.

## Highlights

• We theorise racism as a fundamental cause of ethnic inequalities in vaccine hesitancy.
• We operationalise racism into institutional, community, and individual-level factors.
• Institutional and community-level factors explained the largest part of the inequality.
• Addressing institutional and community-level factors may reduce ethnic inequalities.
• This includes addressing racism, the fundamental cause of ethnic inequalities.

## 1 Introduction

Vaccination against the severe acute respiratory syndrome coronavirus 2 (SARS-CoV-2) is crucial to prevent morbidity and mortality from coronavirus disease (COVID-19) (Henry et al., 2021). Rates of vaccine uptake have not been equal across ethnic groups in the UK (MacKenna et al., 2021; Robertson et al., 2021), which could further exacerbate ethnic inequities in COVID-19 related outcomes (Nazroo & Becares, 2020). Ethnic inequities in vaccine uptake may be the result of unequal and insufficiently tailored distribution of vaccines (Corbie-Smith, 2021), and greater vaccine hesitancy among some minoritised ethnic groups (Freeman et al., 2020; Robertson et al., 2021; SAGE 2020; Woodhead et al., 2021, pp. 1–20; Woolf et al., 2021). Explanations offered for ethnic inequities in vaccine hesitancy have been wide-ranging and have mainly focused around differences in the level of concern about side effects (ONS 2020) and in lack of trust in the development and efficacy of vaccines (Robertson et al., 2021).

**Fig. 1:**
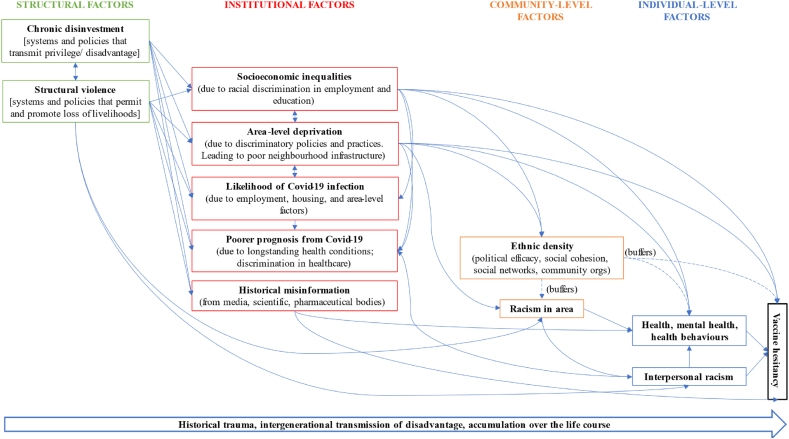
Theoretical Framework detailing the role of racism in leading to ethnic inequalities in vaccine hesitancy.

**Fig. 2:**
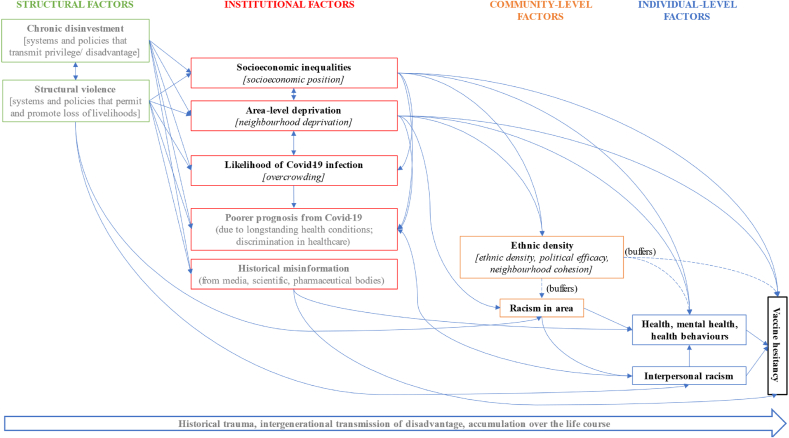
Measurement model detailing the variables captured in the UKHLS to examine theoretical framework.

Here we propose that racism is the fundamental cause of ethnic inequities in vaccine uptake (and other COVID-19 related outcomes). We investigate this in relation to vaccine hesitancy using a theoretical framework that details the mechanisms by which racism at the structural, institutional, and interpersonal level, leads to higher vaccine hesitancy among minoritised ethnic groups (see Fig. 1). We use data from the UK Household Longitudinal Study (UKHLS) to empirically examine these pathways (see Fig. 2 for measurement model).

### 1.1 Racism as the fundamental cause of vaccine hesitancy in minoritised ethnic groups

Racism is a complex system of structuring opportunity and assigning relative value based on phenotypic characteristics, unfairly advantaging some ethnic groups and disadvantaging others (Jones, 2000). Racism manifests on multiple levels including structural, institutional, interpersonal, and internalised (Jones, 2000). Structural racism refers to the “the totality of ways in which societies foster [racial] discrimination, via mutually reinforcing [inequitable] systems … (eg, in housing, education, employment, earnings, benefits, credit, media, health care, criminal justice, etc) that in turn reinforce discriminatory beliefs, values, and distribution of resources” (Bailey et al., 2017). Driven by historical and ongoing processes of colonialism, slavery, and apartheid, structural racism consists of material, cultural, and ideological dimensions (Essed, 1991; Nazroo et al., 2020). Institutional racism is embodied in discriminatory policies and norms embedded in institutional structures and captures a broad array of practices that perpetuate differential access to goods, services, and opportunities within institutions (Jones, 2000; Karlsen & Nazroo, 2002). Interpersonal or personally-mediated racism refers to the everyday expressions of racism, either intentionally or by omission, and can manifest as micro-aggressions, scapegoating, and dehumanisation, including verbal and physical assaults (Jones, 2000). Internalised racism is the acceptance by members of minoritised ethnic groups of negative messages about their own abilities and intrinsic worth (Jones, 2000).

There is a large amount of literature reporting the first-hand experiences of pervasive and lifelong experiences of racial discrimination by minoritised individuals (Essed, 1991; Harris et al., 2006; Katikireddi et al., 2021), and the detrimental association of these experiences on health (Paradies, 2006; Paradies et al., 2015; Williams et al., 2019). Experiences of racial discrimination occur across different stages of the life course (Bécares et al., 2015; Bécares & Zhang, 2018; Essed, 1991; Nuru-Jeter et al., 2008), and across domains such as education, employment, housing, and health care (Bécares et al., 2009; Ben et al., 2017; Wallace et al., 2016). Our theoretical framework, presented in Fig. 1, illustrates the mechanisms through which racism at the structural, institutional, interpersonal levels leads to vaccine hesitancy. It considers community-level factors in patterning vaccine hesitancy, and acknowledges the role of time in perpetuating inequities created by racism through its persistence in historical trauma and in the intra- and intergenerational transmission of disadvantage (Gee et al., 2019).

#### 1.1.1 Structural level

At the structural level, we theorise structural racism to lead to structural violence and chronic disinvestment to maintain the racial order (Bonilla-Silva, 1997) and ensure production and continued reproduction of ethnic inequities broadly, and in vaccine hesitancy as specifically applied to this work. Structural violence is “the cause of the difference between the potential and the actual, between what could have been and what is, and that which impedes the decrease of this distance” (Galtung, 1969, p. 168). In our theoretical model, structural violence is manifested in the invisible systems and processes that permit, promote, and benefit from, the loss of minoritised ethnic livelihoods. The intricate and effective foundations of structural racism support and give rise to institutionalised and individualised practices in subtle, invisible ways leading to what Bonilla-Silva refers to as ‘racism without racists’ (Bonilla-Silva, 2006). Structural violence is embedded into social, political, legislative, and economic systems leading to, and being reinforced by, chronic disinvestment in minoritised ethnic communities across a wide range of intersected systems related to public planning and the built environment, housing, education, employment, criminal justice, health care, and media, all of which have had a role in structuring the stark ethnic inequities in COVID-19 related outcomes (Nazroo & Becares, 2020).

Practices of structural racism are rendered invisible through their gradual enactment over time and enable racism at the institutional level, which in turn amplifies the impact of structural racism, resulting in stark ethnic inequities in socioeconomic outcomes at the individual and neighbourhood levels. Structural racism in the UK is so efficient in its invisible operation that a recent governmental investigation into race and ethnic disparities found no evidence of its existence, concluding instead that socio-economic background (alongside other racialised explanations like family influence, culture, and religion) has a more significant impact on the life chances of minoritised ethnic groups than racism (CRED 2021). The denial of racism (in its structural and other forms) and misplacement of root causes of ethnic health inequities in more proximal determinants such as socioeconomic factors, disregarding the role of racism in leading to socioeconomic inequalities, is a common narrative among academics and political commentators in the UK, and has been central to explanations for ethnic inequities in COVID-19 related outcomes (but see also (Nazroo & Becares, 2020) for contesting arguments).

As shown in Fig. 1, we argue that structural racism, via structural violence and chronic disinvestment, has shaped the landscape of risk (Kelly, 2005) for higher vaccine hesitancy among minoritised ethnic groups by influencing discriminatory policies and practices, and producing and maintaining inequities across institutional factors and enabling racism at the community level, as described below.

#### 1.1.2 Institutional level

The processes of structural racism and their operationalisation via structural violence and chronic disinvestment lead to racism being embedded in institutions’ discriminatory policies and practices. Institutional racism perpetuates differential access to goods, services, and opportunities within institutions (Jones, 2000), resulting in several institutional-level outcomes related to ethnic inequalities. In our theoretical model, these are represented by socio-economic inequalities, area-level deprivation, likelihood of coronavirus infection, and historical misinformation. These institutional-level factors are interrelated and maintain and strengthen each other over time and across generations.

Studies on vaccine hesitancy have documented the role of socio-economic factors at the individual and area-level in patterning inequities (Bertoncello et al., 2020; MacKenna et al., 2021; Murphy et al., 2021; Robertson et al., 2021; Viswanath et al., 2021; Woolf et al., 2021). Studies that have examined other COVID-19 related outcomes, including infection and mortality, have found similar associations between socioeconomic disadvantage and poorer outcomes (Mathur et al., 2021). These studies have centred the role of socio-economic disadvantage as the primary cause of vaccine hesitancy or other COVID-19 outcomes, without considering the systems and processes *disadvantaging* minoritised ethnic groups. These arrows are explicit in our model. As we described earlier, racism (both structural and institutional) patterns both ethnic inequities in socioeconomic outcomes and area-level deprivation. Institutional racism in one sector or domain (e.g., education, the criminal justice system, urban planning) reinforces it in other sectors (e.g., employment, housing), forming a large, interconnected system that produces and maintains ethnic inequities (Bailey et al., 2017). Our theoretical model therefore emphasises the role of institutional racism, and the discriminatory policies and practices it enables, produces, and promotes, in patterning socioeconomic inequities and area-level deprivation, which are relevant for vaccine hesitancy. For example, historic racist housing policies and practices have discriminated against minoritised ethnic groups, leading to ethnic inequities in access to social and private housing sectors. These policies and practices, including discriminatory processes in the social housing system, discrimination by private landlords in selecting tenants, ‘racial steering’ by estate agents, and restricted access to mortgage lending (Bazargan, Cobb, Assari, & Bazargan-Hejazi, 2022; Jeffers and Hoggett, 1995; Phillips and Harrison, 2010; Harrison and Stevens, 1981; Robinson, 2002), compounded by historical and current disinvestment in minoritised ethnic communities, have resulted in the disproportionate representation of minoritised ethnic minority groups in the most deprived neighbourhoods (Jivraj & Khan, 2015). Residence in neighbourhoods with concentrated poverty and chronic disinvestment affects access to and quality of public services, including education and healthcare, contributing to limited opportunities for upward social mobility, and leading to long-term detrimental impacts on health (White & Borrell, 2011; Williams & Collins, 2001). The structure and workings of the housing market and the educational system in the UK is such that discrimination and differential access to housing for minoritised ethnic groups is related to unequal access to the best performing and most desirable state schools, with implications for ethnic inequities in education and employment (Lymperopoulou & Finney, 2017; Feng et al., 2015).

We therefore propose in our theoretical model a direct link between structural violence and chronic disinvestment, and the resulting inequalities in socioeconomic indicators (income, education, employment) and area-level deprivation (e.g., substandard levels of housing stock, limited transport, increased pollution, reduced access to green space, geographical barriers) that have been associated with increased vaccine hesitancy and poor uptake rates. Area deprivation may have also resulted in additional barriers to accessing centralised vaccination sites for some minoritised ethnic groups, for example in terms of journey time and cost (Watkinson et al., 2022).

We place increased likelihood of coronavirus infection and poorer prognosis from infection at the institutional level because, although these are measured at the individual-level, the stark ethnic inequities documented in these outcomes are a manifestation of, and result from, institutional-level factors, including socioeconomic inequities at the individual and area levels (Nazroo & Becares, 2020), and differential healthcare quality enabled by racism (Sjoding et al., 2020).

Another key construct that we situate at the institutional level is historical misinformation leading to mistrust. Studies show that participants who report increased hesitancy are also more likely to report mistrust in government officials, scientists, and health care professionals (Bhanu et al., 2021; Hassan et al., 2021; Lindholt et al., 2021; Murphy et al., 2021; Woodhead et al., 2021, pp. 1–20; Woolf et al., 2021). For minoritised ethnic groups, mistrust in these institutions arises from a legacy of abuses in research, experiences of unfair treatment in healthcare, pernicious media misinformation (including social media) (Bazargan, Cobb, Assari, & Bazargan-Hejazi, 2022; Henderson et al., 2014), and governmental responses to events that have detrimentally impacted minoritised ethnic communities in the UK, such as the Grenfell Tower catastrophe and the Windrush Scandal (Ateghang-Awankem & Yongabi Anchang, 2021; Woodhead et al., 2021, pp. 1–20).

#### 1.1.3 Community-level

At the community level we consider the health-promoting and protecting effects that living in diverse communities has for the health and health behaviours of minoritised ethnic groups, including with relation to vaccine hesitancy. A large body of research has shown that once the concentration of poverty and disadvantage in the neighbourhood has been adjusted for, the residential concentration of ethnic minority people, or ethnic density, has been associated with protective effects on health and health behaviours, a so-called ethnic density effect (Bécares et al., 2012, 2017; Halpern & Nazroo, 2000; Shaw et al., 2012). Positive health outcomes associated with ethnic density are attributed to the protective and buffering effects from the direct or indirect consequences of discrimination and racial harassment (Bécares et al., 2009, 2012). Other mechanisms include enhanced social cohesion, mutual social support, a stronger sense of community and belongingness, and increased political efficacy (Bécares & Das-Munshi, 2013; Bécares et al., 2011, 2013; Lindholt et al., 2021). Ethnic density may be a protective factor against vaccine hesitancy because of the increased social capital and social cohesion that it fosters (Bécares et al., 2011). Promotion of vaccination by members of trusted networks, and involvement of voluntary, faith, community, and charity organisations in vaccination efforts, has improved vaccine confidence and uptake rates among minoritised populations (Lott et al., 2020).

#### 1.1.4 Interpersonal level

At the interpersonal level, the key construct patterning ethnic inequalities in vaccine hesitancy is direct or vicarious experiences of racist events, which mainly operate by threatening ontological security leading to poor mental and physical health (Paradies, 2006; Paradies et al., 2015; Williams et al., 2019), which have been associated with increased vaccine hesitancy (Lorenz et al., 2013). Racism structures individual risk (Katikireddi et al., 2021), and our theoretical model includes only two constructs at the individual level to reflect that, despite being more visible and empirically operationalisable at the individual level, the foundational causes of vaccine hesitancy are located at institutional and structural levels.

## 2 Material and methods

We test our theoretical model using nationally representative data from the UK Household Longitudinal Study (UKHLS). Due to the limitations of secondary data analyses we are not able to empirically examine all variables or levels hypothesised in our theoretical model, but we can explore how the majority of constructs proposed are associated with ethnic inequities in vaccine hesitancy. Although we can't empirically examine variables that measure structural racism, we can capture related constructs at the institutional, community, and individual-level. Fig. 2 presents the constructs available in the empirical model (in bold), and an indication of the measured variable.

### 2.1 Data

Data come from Wave 6 of the UKHLS COVID-19 Survey (University of Essex, 2021), which was carried out during the initial stages of the UK vaccination programme, from 24th November to December 1, 2020. In 2020, during the COVID-19 pandemic, participants of what were then the two most recent waves, Waves 8 or 9, of UKHLS, which were collected between 2016 and 2019, were invited to take part in the COVID-19 Survey either online or by phone. Initial web surveys were carried out from April to July, with follow up surveys carried out every two months. Participants were eligible for the Wave 6 online COVID-19 Survey if they had participated in any of the prior COVID-19 Surveys. Variables to operationalise structural aspects of racism were also taken from the most recent pre-pandemic UKHLS waves (Wave 8 & 9), and key sociodemographic measures, ethnicity, and country of birth, were taken from the first wave when participants joined UKHLS, which started in 2009. From this, we derived two analytic samples. First, 7759 people aged over 18 who were resident in England and had complete data for all variables used to operationalise structural racism in our theoretical model. The sample was restricted to England because area deprivation and the census data used to calculate ethnicity density are measured differently for the other UK countries. Second, 1182 people from minoritised ethnic groups who had completed an additional interview that included questions on their experience of racism in Wave 9.

Ethical approval was granted by the University of Essex Ethics Committee for all the UKHLS main Study waves and the COVID-19 surveys (ETH 1920-1271). No additional ethical approval was necessary for this secondary data analysis.

#### 2.1.1 Ethnicity

Ethnicity was self-reported by participants when they were first interviewed for the study by using the 2011 Census question, which includes 18 different categories. The two largest ethnic groups, white British and Indian, were kept as distinct groups. Other groups were aggregated as follows: Pakistani or Bangladeshi; Black (including Caribbean, African and Other Black); Other White; Mixed; Other Asian; and Other ethnicity. While there is some degree of heterogeneity within these groups, they were aggregated into theoretically coherent groups in order to provide adequate numbers for statistical analysis.

#### 2.1.2 Vaccine hesitancy

We assessed vaccine hesitancy (Very likely or Likely/Unlikely or very unlikely) with a single question. “Imagine that a vaccine against COVID-19 was available for anyone who wanted it? How likely or unlikely would you be to take the vaccine?”

#### 2.1.3 Explanatory variables

We grouped variables as either operationalising the pathways linked to racism at the different levels mapping out as closely as possible to the theoretical framework, depending on the availability of variables (institutional, community, or individual; see Fig. 2 for measurement model), or as demographic characteristics.

1. Institutional-level

We used four measures of socioeconomic position (SEP) based on data collected in the November COVID-19 Survey. Subjective financial situation (living comfortably/doing alright/just about getting by/finding it quite difficult or very difficult) was assessed using a single question “How well would you say you yourself are managing financially these days?” Car use (At least once a day/Less than once a day but at least 3 times a week/once or twice a week/less than that or never) was assessed by asking how frequently participants travelled by private car or van, either as a driver or passenger. Car use, in addition to acting as an indicator of SEP, may be an indicator of participants' ability to travel to vaccination centres. Housing Tenure (own outright/own with a mortgage/socially rented/private renting) was assessed based on responses in Wave 9. Education (degree/A-level or equivalents/GCSE or equivalents/none) was derived from participants’ responses across all waves of UKHLS.

Area deprivation was assessed using deciles from the 2019 Index of Multiple Deprivation (McLennan et al., 2019) for Lower Super Output Areas (LSOA) in which participants were resident at the time of the survey.

Overcrowding (under-occupied/balanced/overcrowded) was assessed by comparing the number of bedrooms in the house to the number of people (Cable & Sacker, 2019). Households were classified as under-occupied if they had more bedrooms than needed, balanced if bedrooms matched the number of people, or overcrowded if the total was exceeded.

2. Community-level

We assessed community-level factors using three measures from the November COVID-19 Survey. Ethnic density was calculated as the proportion of non-White British people in the English 2011 Census for each participants' area of residence measured at the LSOA level. Neighbourhood cohesion was assessed using a continuous measure created by summing the responses measured on a 5-point Likert scale (1 – strongly agree, 5 strongly disagree) to 5 items indicating interactions between people in their neighbourhood, such as “People around here are willing to help their neighbours.” Internal political efficacy, which relates to one's own competency in understanding and participating effectively in politics, and external political efficacy, which refers to beliefs about the responsiveness of government authorities to citizen's demands, (both continuous) were assessed with four items from a widely used measure of political efficacy (Niemi et al., 1991) recorded in Wave 9.

We assessed racism in the participant's area (not at all common/not very common/fairly common/very common) with a single item “How common in your area are insults or attacks to do with someone's race or colour”?

3. Individual-level

The UKHLS asked about experiences of interpersonal racism to participants who completed an additional questionnaire for wave 9. The measure of experienced racial discrimination used in the analyses is an adaptation from one used in a previous study (Wallace et al., 2016) and was assessed through two sets of questions. The first set of questions asked participants whether they: (1) had felt unsafe; (2) had avoided going to or being in several locations; (3) had been insulted, called names, threatened, or shouted at; or (4) had been physically attacked in the last 12 months; for each of a set of locations including at school, at college, at work, on public transport, outside, in a public place or at home. If participants responded affirmatively, they were then asked to specify the possible reasons for the discrimination from the following attributions: sex, age, ethnicity, sexual orientation, health or disability, nationality, religion, language or accent, or dress or appearance. The total number of exposures to racism was calculated as the number of times that people reported being attacked because of their ethnicity, nationality, or religion. Participants were also asked whether they had been turned down for a job, and if so whether it was because they had been discriminated against for any of the reasons above. This was added to the measure, which was then converted into a categorical variable coded as follows: zero, one, and two or more experiences of racism.

Five indicators of health, including measures of health behaviour, physical, mental and social wellbeing (World Health Organization (WHO), 1948) were drawn from the November COVID-19 survey. Clinical vulnerability to serious illness from COVID-19 (no risk/moderate risk/high risk) was assessed in accordance with NHS guidelines (Institute for Social and Economic Research, 2021). Participants were classified as to whether they were vulnerable based on any treatment or conditions that they had and whether they were aged over 70 years. Self-rated health (excellent or very good/good/fair or poor) of participants was assessed with the question “In general, would you say your health is …” Loneliness (hardly ever or never/some of the time/often) was assessed using a single item “In the last 4 weeks, how often did you feel lonely?” (HM Government, 2018). Life satisfaction was assessed using a continuous measure defined by participant's responses on a seven-point Likert scale (completely dissatisfied to completely satisfied) to the question “How satisfied are you currently with your life overall?” Psychological distress was assessed using a continuous measure calculated from the 12 Item General Health Questionnaire (Goldberg et al., 1997). Two additional health indicators were drawn from Wave 9; limiting longstanding illness (yes/no) was assessed by asking participants if they had a longstanding physical or mental impairment, illness or disability that had either troubled them for a least 12 months or was likely to trouble them for more than 12 months. Smoking (yes/no) was assessed by a single item asking whether participants smoked cigarettes.

#### 2.1.4 Demographic variables

Five demographic variables were based on data from the November COVID-19 survey. Age in years was calculated based on date of birth and the date when participants completed the survey. Gender was based on the participant's most recent survey response. Other demographic variables included partnership status, living with school-aged children, living with a person over 70, and country of origin. Partnership status (yes/no) was assessed based on whether participants reported one of the following relationships: husband, wife, civil partner, partner, or cohabitee. Living with school-aged children (yes/no) was based on cohort members reporting living with any household members between the age of 5 and 18. Living with person over 70 (yes/no) was based on cohort members living with another person of the appropriate age. In addition, country of origin (born in UK/not born in UK) was assessed when participants first joined UKHLS.

### 2.2 Analytical approach

The analytical plan is divided into three sets of analyses, as described below. The first two sets of analyses used the main analytic sample of people resident only in England (n = 7759). The third focused on the people from minoritised ethnic groups who were asked additional questions on their experiences of racism and discrimination at Wave 9 (n = 1182). Data were prepared and analysed using Stata 16.1. Graphs were created using the ggplot 2 package in R. Participants who had zero cross-sectional weights for Wave 9 of UKHLS were also zero weighted for the COVID-19 web surveys. This affected comparatively high proportions of the participants from minoritised ethnic groups (21.5% for Pakistani Bangladeshi, 19.2% for Black and 18.2% for Indian). Given the comparatively small sample size of some minoritised ethnic groups this would have had a deleterious impact on power, so weights were not used for these analyses, which aim to estimate causal pathways rather than estimate characteristics of the population from which the sample is drawn. Our final regression models included a wide range of covariates, covering those that would be used to create sample weights, so parameter estimates from these models were likely to be very similar to those for weighted models.

#### 2.2.1 Ethnic inequalities in vaccine hesitancy before and after adjusting for age and gender

In the first set of analyses we used logistic regression models to assess the relationship between vaccine hesitancy and ethnicity before and after adjusting for age (operationalised using both linear and quadratic terms) and gender. Following this, the margins command in Stata (Williams, 2012) was used to calculate the predicted probability of vaccine hesitancy before and after adjustment. Subsequent to this, we used the Karlson–Holm–Breen (KHB) method (Karlson et al., 2012) implemented within Stata and accounting for clustering at LSOA to formally compare the relationship between ethnicity and vaccine hesitancy before and after adjusting for age and gender. The KHB method was required because one of the limitations of logistic regression modelling is that introducing covariates into a model may alter and rescale, due to change in the residual variance, the relationship between the exposure and the outcome, even if the covariate does not confound the relationship between the exposure and outcome (Karlson et al., 2012; Vittinghoff et al., 2012). The KHB method addresses this problem, by comparing regression coefficients for the exposure from two models (one including the exposure and covariates, and the second including the exposure and residualised version of the covariate), to show the true impact of the covariates. Using the KHB method we calculated both average partial effects (APEs) and odds ratios for vaccine hesitancy, before and after adjustment. In addition, based on the APEs we calculated the percentage change in ethnic inequalities on adjustment for age and gender by dividing the difference in APEs between the adjusted and unadjusted models by the APE for the adjusted model.

#### 2.2.2 Ethnic inequalities in vaccine hesitancy before and after adjusting for domains of racism at institutional and community level

The second set of analyses investigated the proportion of ethnic inequalities in vaccine hesitancy potentially explained by each of the domains of racism at the institutional and community-level once both age and gender had been accounted for. This was carried out using models derived using the KHB Method. This time the KHB method was used to calculate and compare APEs and odds ratios for ethnic inequalities in vaccine hesitancy models adjusting for age and gender (baseline) with models including variables from each domain of racism in turn, other demographic factors, and a final model including all variables. From APE models, the percentage of ethnic inequality explained by each domain was calculated and presented graphically.

#### 2.2.3 Individual exposure to racism and vaccine hesitancy

The relationship between interpersonal racism and vaccine hesitancy was investigated in the subsample of people with minoritised ethnic backgrounds who completed the questions related to the experience of racism in the additional questionnaire from Wave 9 of UKHLS. The third set of analyses does not follow the same approach as the prior analyses because the sample size completing the additional questionnaire was greatly reduced. So, in a model adjusting for age, gender, and ethnicity we calculated the conditional odds ratio for vaccine hesitancy by experiences of interpersonal racism.

## 3 Results

The main analytic sample is described in Table 1. Overall, just under 15% of the sample said they were unlikely or very unlikely to take a COVID-19 vaccine. Approximately 14% of the sample were from a minoritised ethnic group. Only 9.6% of the sample had been born outside the UK. The sample was more socioeconomically advantaged than the general population, as the mean decile of area deprivation was 6.2 and 48.7% of the sample had studied to at least degree level.

**Table 1:** Descriptive statistics for the main analytic sample of the UKHLS.

Variable	N	%	Variable	N	%
*Outcome*			*Institutional*		
**COVID19 Vaccine Hesitancy**			**Education**		
Not Hesitant	6624	85.4	Degree	3775	48.7
Hesitant	1135	14.6	A level	841	10.8
			GCSE	1993	25.7
*Main exposures*			None	1150	14.8
**Ethnicity**			**Subjective financial situation**		
Black	168	2.2	Comfortably	2527	32.6
Indian	219	2.8	Doing alright	3688	47.5
Mixed	125	1.6	Just about getting by	1198	15.4
Other Asian	92	1.2	Difficult or Very difficult	346	4.5
Other Ethnicity	35	0.5	**Housing Tenure**		
Other White	265	3.4	Own outright	3392	43.7
Pakistani and Bangladeshi	174	2.2	Own with a mortgage	3086	39.8
White British	6681	86.1	Socially rented	609	7.9
			Private rented	651	8.4
*Core demographic variables*			Other	21	0.3
**Gender**			**Overcrowding**		
Male	3256	42.0	Under-occupied	6510	83.9
Female	4503	58.0	Balanced	934	12.0
			Overcrowded	315	4.1
*Other demographic variables*			**Access to car**		
**Country of origin**			At least once a day	2234	28.8
Born in UK	7016	90.4	less than once a day by to three times a week	1896	24.4
Not born in UK	743	9.6	Once or twice a week	2338	30.1
**Partnership status**			Less than that or never.	1291	16.6
Yes	5610	72.3			
No	2149	27.7	*Community level variables*		
**Presence of school age children**			**Area Racism**		
None	5993	77.2	Not at all	5146	66.3
One or more	1766	22.8	Not very	2401	30.9
**Household contains person over 70**			Fairly or very common	212	2.7
Zero	6446	83.1			
One or more	1313	16.9	*Continuous measures*		
				Mean	SD. Dev.
*Health variables*			**Age**	55.4	15.5
**Clinical vulnerability**			**Decile of deprivation**	6.2	2.7
No Risk	4348	56.0	**Neighbourhood cohesion**	18.6	3.2
Moderate Risk	2912	37.5	**Internal political efficacy**	6.1	1.9
High risk	499	6.4	**External political efficacy**	5.6	1.8
**Self-rated health**			**Proportion of LSOA Not white British**	0.16	0.20
Excellent/Very good	4155	53.6	**GHQ**	12.5	5.9
Good	2526	32.6	**Life Satisfaction**	3.0	1.5
Fair or poor	1078	13.9			
**Limiting long standing illness**					
No	4978	64.2			
Yes	2781	35.8			
**Loneliness**					
Hardly ever or never	4886	63.0			
Some of the time	2422	31.2			
Often	451	5.8			
**Smoking**					
No	7114	91.7			
Yes	645	8.3			

### 3.1 Ethnic inequalities in vaccine hesitancy

**Fig. 3:**
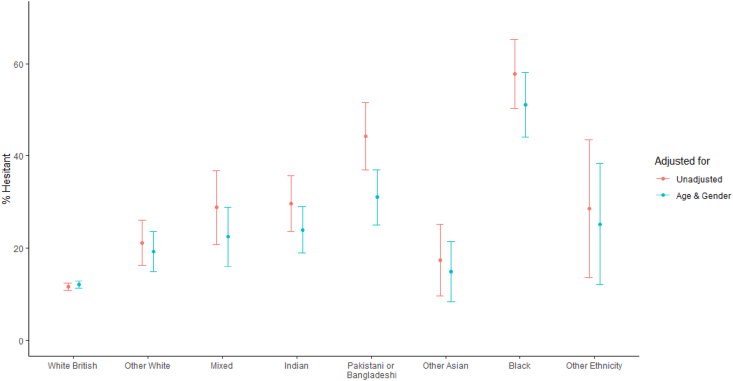
Percent of COVID19 vaccine hesitancy by ethnic group both in unadjusted models and adjusting for age and sex.

Fig. 3 presents the probabilities of vaccine hesitancy before and after adjusting for age, age squared, and gender. Unadjusted vaccine hesitancy ranged from 12% for white British people to 56% for Black people. Adjusting for age and gender reduced the difference in vaccine hesitancy between the white British people and nearly all ethnic groups (see Fig. 3 and Supplementary Table 1). Based on APEs generated using the KHB method, the percentage of ethnic differences in vaccine hesitancy explained by age and gender relative to white British people was 15% for Black, 24% for Other ethnicity, 28% for Other white, 37% for Indian, 43% for Mixed ethnicity, 44% for Pakistani or Bangladeshi, and 62% for Other Asian.

**Fig. 4:**
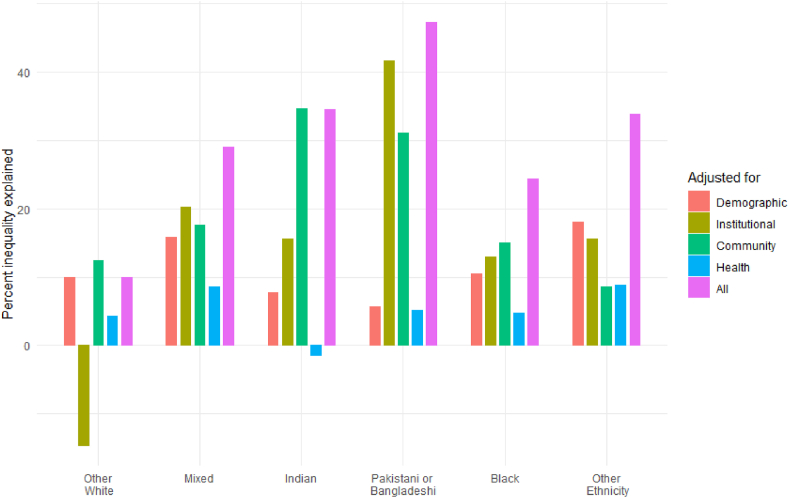
Percent of ethnic inequalities in COVID19 vaccine hesitancy explained by each domain^1^ using APEs derived from KHB regression models after adjusting for age and gender. 1. Demographic variables are: Country of origin, Partnership status, Presence of school age children, and Household containing person over 70. Institutional variables are Education, Subjective financial situation, Tenure, Overcrowding, Area deprivation, and Access to car. Community level variables are: Neighbourhood cohesion, Internal political efficacy, External political efficacy, Area racism, and Ethnic density. Health variables are: Clinical vulnerability, Self-rated health, Limiting longstanding illness, GHQ-12, Life satisfaction, and Smoking.

Fig. 4 shows the percentage of APEs (Supplementary Table 3 shows the APEs; Supplementary Table 4 shows the Odds Ratios) for vaccine hesitancy explained by age, gender, demographic characteristics, and each of the pathways linked to racism: institutional, community, individual, and all domains combined. The demographic variables (country of origin, partnership status, presence of an older person in the household) explained a modest percentage of inequalities of vaccine hesitancy among minoritised ethnic groups, ranging from 5.6% for Pakistani or Bangladeshi people, to 18.1% for Other ethnicities. The percentage of institutional factors in explaining vaccine hesitancy varied across groups, explaining 41.6% of the difference for Pakistani or Bangladeshi people, 20.2% for Indian people, and 12.9% for Black people. The community-level pathway was an important explanatory factor, explaining more than 30% of the ethnic inequality in vaccine hesitancy for Indian, and Pakistani or Bangladeshi people, and being the most important explanatory pathway for the Black group. The individual-level pathways (capturing health measures) were relatively unimportant in explaining vaccine hesitancy, explaining at most only 8.6% of the difference (for the mixed ethnic group). Finally, the model containing all variables explained a reasonably high proportion of ethnic inequalities for nearly all ethnic groups, apart from the Other White ethnic group. The highest proportion of inequality potentially explained was for Pakistani or Bangladeshi people (47.2%), with around a third for Indian and a quarter for Black people. The full model explained less than 10% of the ethnic inequality in vaccine hesitancy for members of the Other White group.

The odds ratios for each variable included in the final model are shown in Supplementary Table 5. Particularly important variables include both measures of political efficacy, neighbourhood cohesion, education, subjective finances and area deprivation.

### 3.2 Interpersonal racism and vaccine hesitancy

For minoritised ethnic participants who had completed the racism questions in Wave 9 of UKHLS, we investigated if experience of interpersonal racism (individual-level pathway) was associated with vaccine hesitancy. Prevalence of experienced racial discrimination was comparatively low, with 8.8% of the sample reporting experiences of racial discrimination once, and 9.2% two or more times. In a model adjusting for age, gender, and ethnicity there was no evidence that being exposed to one domain of interpersonal racism (OR 0.66 95% CI 0.42 to 1.05) or two or more domains (OR 0.96 95% CI 0.62 to 1.50) was associated with increased vaccine hesitancy (See Supplementary Table 7).

## 4 Discussion

This paper proposes a theoretical framework that argues for the fundamental role of racism in leading to ethnic inequities in vaccine hesitancy, and tests this framework using data from the UK Household Longitudinal Study. Our findings show that factors at the institutional-level explained the largest part (42%) of the inequality for Pakistani or Bangladeshi people, and community-level factors were the most important factors for Indian and Black groups, explaining 35% and 15% of the inequality, respectively. The individual-level pathway was relatively unimportant in explaining vaccine hesitancy for all groups. We found that age and sex explained a large percentage of ethnic inequalities across groups, as has been previously reported (Katikireddi et al., 2021; Liu and Li, 2021).

These findings are closely related to studies in other countries that have shown an association between experiences of racial discrimination and increased vaccine hesitancy (Savoia et al., 2021), as well as to research that has examined associations between different manifestations and outcomes of structural racism and other COVID-19 related outcomes. These studies have shown associations between residential segregation and the consequences of social distancing (White et al., 2021); racism in employment settings and the disproportionate impact of the pandemic on ethnic minority health care workers (Ramamurthy et al., 2022); and ethnic inequities in arrest and incarceration rates and increased COVID-19 incidence in minoritised ethnic communities (Reinhart & Chen, 2021).

In our theoretical model we aimed to specify the insidiousness of structural racism in embedding ethnic inequities in laws and policies across interconnected systems and institutions in the UK, which created a landscape of risk for the observed ethnic inequities in COVID-19 vaccine hesitancy. The limited availability of data existent to test our theoretical model meant that in our empirical model we were restricted by measures capturing constructs that directly result from, but are operationally different from, structural racism. Recent studies in the US have empirically documented the association between structural racism and health inequities using individual exposure measures like police encounters (Theall et al., 2022) and racialised disenfranchisement (Homan & Brown, 2022), or multidimensional measures of structural racism developed using latent variable models (Chantarat et al., 2021). Although we didn't have access to similar constructs, measures we had available at the institutional-level such as inequities in socioeconomic position, area-level deprivation, and household overcrowding, are clear outcomes of structural and institutional racism. We find these explain a large percentage of ethnic inequities in vaccine hesitancy, particularly among Bangladeshi and Pakistani ethnic groups.

The experiences and circumstances of different minoritised ethnic groups are distinct, and this is reflected in our findings. For example, in terms of socioeconomic position Pakistani and Bangladeshi people in the UK are the most disadvantaged of all the ethnic groups analysed here (Bazargan, Cobb, Assari, & Bazargan-Hejazi, 2022), so it is expected that institutional-level factors including socioeconomic position and area-level deprivation would explain a large percentage of inequality in vaccine hesitancy for them. Our methodological approach allowed us to explore multiple ethnic groups and consider heterogeneity appropriately. The use of the KHB method addresses the issues of non-collapsibility of odds ratios and enables ethnic inequalities to be compared between models with different levels of adjustment. The incorporation of an ethnic minority boost sample into the UKHLS enabled analysis of multiple ethnic groups, although we did have to combine Pakistani with Bangladeshi people, and Black Caribbean with Black African people, into the same groups because of small sample sizes of the individual ethnic groups. We combined these groups following sensitivity analyses comparing relative similarity of profiles across individual ethnic groups to ensure theoretical and methodological robustness of groupings. The proportion of APEs for each ethnicity explained by adjusting for each domain of variables is relative to the size of initial ethnic inequalities, and the size of the initial ethnic inequality should be born in mind before interpreting the proportion of APEs explained.

Limitations of our study relate mainly to the constructs we were able to measure in the UKHLS. We didn't have access to measures of structural racism as described above. We also lacked data for some other theoretical pathways, such as historical misinformation from the media, and better evidence for this might be collected using qualitative methodology. The measure of interpersonal racism recorded experiences in the 12 months preceding wave 9 of UKHLS, other experiences of interpersonal racism not measured in the domains captured here, or exposure to racism in different points in the life course, may have shaped views relating to vaccine uptake, but we are unable to capture this relationship. We also had imperfect measures for some of the observed constructs. Despite these limitations, our measurement model explained a large proportion of the inequalities in vaccine hesitancy.

Our findings suggest that if policy intervened on key institutional and community-level factors, considerable success in reducing ethnic inequalities might be achieved. These factors that can be modified by policy are not related to individual-level choices or behaviours – a focus of most nudging-style policy in the UK – but rather are factors shaped by structural and institutional racism, as described in our theoretical model. Short to mid-term policies that aim to redress ethnic inequities in vaccine hesitancy, or other COVID-19 related outcomes, should therefore focus on key institutional and community determinants of health, including reducing inequalities in education, housing tenure, area-level deprivation, or overcrowding. In the long term, focusing on these factors alone will not suffice. Addressing the production and reproduction of ethnic inequities, and dismantling the racist structures and systems that reproduce and maintain these inequities, require changing laws, policies, and practices in ways that produce sustained or fundamental change (Braveman et al., 2022).

## Financial disclosure

This study was funded by the Economics and Social Research Council (ES/W000849/1). SVK acknowledges funding from a NRS Senior Clinical Fellowship (SCAF/15/02). RJS, SA and SVK acknowledge funding from the Medical Research Council (MC_UU_00022/2) and the Scottish Government Chief Scientist Office (SPHSU17).

## CRediT author statement

Laia Bécares: Conceptualization, Writing - original draft, Writing – review & editing, Visualization, Supervision, Project administration. Richard J. Shaw: Methodology, Software, Formal analysis, Data curation, Writing - original draft, Writing – review & editing. Srinivasa Vittal Katikireddi: Supervision, Writing – review & editing.Patricia Irizar: Writing – review & editing. Sarah Amele: Writing – review & editing. Dharmi Kapadia: Writing – review & editing. James Nazroo: Writing – review & editing. Harry Taylor: Writing – review & editing.

## Ethical statement

Ethical approval was granted by the University of Essex Ethics Committee for all the UKHLS main Study waves and the COVID-19 surveys (ETH 1920-1271). No additional ethical approval was necessary for this secondary data analysis.

## Declaration of competing interest

SVK was Co-Chair of the Scottish Government Expert Reference Group on Ethnicity and COVID-19 and a member of the UK Scientific Advisory Group on Emergencies (SAGE) subgroup on Ethnicity.
