# Supplementary material for: Racism as the fundamental cause of ethnic inequities in COVID-19 vaccine hesitancy: A theoretical framework and empirical exploration using the UK Household Longitudinal Study
Source: SSM Popul Health. 2022 Jun 24;19:101150. doi: 10.1016/j.ssmph.2022.101150 (PMC9225926; doi:10.1016/j.ssmph.2022.101150)
Supplement: Multimedia component 1 [file mmc1.docx]

## **Supplementary Tables**

**Supplementary Table 1: Percent of COVID19 Vaccine hesitancy by ethnic group in unadjusted models and models adjusting for age and sex.**

|  | Unadjusted | Adjusted age and sex |
| --- | --- | --- |
|  | Percent  (Confidence interval) | Percent  (Confidence interval) |
| Black | 57.7 (50.3 to 65.2) | 51.1 (44.1 to 58.1) |
| Indian | 29.7 (23.6 to 35.7) | 24.0 (18.9 to 29.0) |
| Mixed | 28.8 (20.9 to 36.7) | 22.5 (16.0 to 28.9) |
| Other Asian | 17.4 (9.6 to 25.1) | 14.9 (8.3 to 21.5) |
| Other Ethnicity | 28.6 (13.6 to 43.5) | 25.2 (12.1 to 38.3) |
| Other White | 21.1 (16.2 to 26.0) | 19.2 (14.8 to 23.7) |
| Pakistani or Bangladeshi | 44.3 (36.9 to 51.6) | 31.0 (25.0 to 37.0) |
| White British | 11.6 (10.9 to 12.4) | 12.1 (11.4 to 12.9) |
|  |  |  |

**Supplementary Table 2: Odds ratios and average partial effects for ethnic inequalities in vaccine hesitancy calculated from KHB regression models before and after adjusting for age and gender.**

|  | Odds Ratio | APE^1^ |
| --- | --- | --- |
|  | OR (95% CI) | Coef (95% CI) |
| **Reference group = White British** |  |  |
| **Black** |  |  |
| Baseline | 12.96 (8.98-18.7)*** | 0.46 (0.38-0.54)*** |
| Adjusting age and gender | 9.14 (6.34-13.17)*** | 0.39 (0.31-0.47)*** |
| Difference between model | 1.42 (1-2)* | 0.07 |
| % explained by age and gender |  | 15.22 |
| **Indian** |  |  |
| Baseline | 3.66 (2.61-5.13)*** | 0.19 (0.13-0.25)*** |
| Adjusting age and gender | 2.41 (1.71-3.38)*** | 0.12 (0.06-0.17)*** |
| Difference between model | 1.52 (1.08-2.15)* | 0.07 |
| % explained by age and gender |  | 37.46 |
| **Mixed** |  |  |
| Baseline | 3.49 (2.31-5.25)*** | 0.18 (0.1-0.26)*** |
| Adjusting age and gender | 2.2 (1.46-3.31)*** | 0.10 (0.04-0.17)** |
| Difference between model | 1.59 (1.12-2.24)** | 0.08 |
| % explained by age and gender |  | 42.5 |
| **Other Asian** |  |  |
| Baseline | 1.83 (1.06-3.17)* | 0.07 (-0.01-0.15)+ |
| Adjusting age and gender | 1.28 (0.74-2.23) | 0.03 (-0.04-0.09) |
| Difference between model | 1.43 (1.01-2.02)* | 0.04 |
| % explained by age and gender |  | 61.71 |
| **Other Ethnicity** |  |  |
| Baseline | 3.34 (1.54-7.28)** | 0.17 (0.03-0.32)* |
| Adjusting age and gender | 2.59 (1.19-5.62)* | 0.13 (0-0.26)+ |
| Difference between model | 1.29 (0.92-1.82) | 0.04 |
| % explained |  | 24.13 |
| **Other White** |  |  |
| Baseline | 2.19 (1.59-3.01)*** | 0.10 (0.05-0.15)*** |
| Adjusting age and gender | 1.78 (1.29-2.45)*** | 0.07 (0.03-0.12)** |
| Difference between model | 1.23 (0.87-1.73) | 0.03 |
| % explained by age and gender |  | 28.43 |
| **Pakistani or Bangladeshi** |  |  |
| Baseline | 7.33 (5.11-10.52)*** | 0.33 (0.26-0.41)*** |
| Adjusting age and gender | 3.55 (2.47-5.09)*** | 0.19 (0.12-0.26)*** |
| Difference between model | 2.07 (1.45-2.94)*** | 0.15 |
| % explained by age and gender |  | 43.55 |

1 KHB regression command does not calculate standard errors for average partial effects.

**Supplementary Table 3: Using average partial effects from KHB regression models to compare ethnic inequalities before adjusting for variables in each domain.**

Coefficients presented are from a reduced model^1^, full model adjusting for variables in each domain, difference between models, and percent of reduced model explained by the difference in models.

| Ethnicity | Demographic^2^ | Institutional^3^ | Community4 | Health^5^ | All |
| --- | --- | --- | --- | --- | --- |
| (Reference White British) | APE (95% CI) | APE(95% CI) | APE (95% CI) | APE (95% CI) | APE (95% CI) |
| **Black** |  |  |  |  |  |
| Reduced | 0.39 (0.31-0.46)*** | 0.39 (0.31-0.47)*** | 0.39 (0.31-0.47)*** | 0.39 (0.31-0.47)*** | 0.39 (0.31-0.46)*** |
| Full | 0.35 (0.27-0.43)*** | 0.34 (0.26-0.42)*** | 0.33 (0.25-0.41)*** | 0.37 (0.29-0.45)*** | 0.29 (0.21-0.38)*** |
| Diff | 0.04 | 0.05 | 0.06 | 0.02 | 0.1 |
| Explained | 10.46 | 12.91 | 15.03 | 4.71 | 24.41 |
| **Indian** |  |  |  |  |  |
| Reduced | 0.12 (0.06-0.18)*** | 0.12 (0.06-0.18)*** | 0.12 (0.06-0.17)*** | 0.12 (0.06-0.18)*** | 0.12 (0.06-0.17)*** |
| Full | 0.11 (0.05-0.17)*** | 0.1 (0.04-0.16)*** | 0.08 (0.02-0.13)** | 0.12 (0.06-0.18)*** | 0.08 (0.02-0.14)** |
| Diff | 0.01 | 0.02 | 0.04 | 0.00 | 0.04 |
| % explained | 7.78 | 15.57 | 34.61 | -1.42 | 34.47 |
| **Mixed** |  |  |  |  |  |
| Reduced | 0.1 (0.04-0.17)** | 0.1 (0.04-0.16)*** | 0.1 (0.04-0.17)*** | 0.1 (0.04-0.17)** | 0.1 (0.04-0.16)*** |
| Full | 0.09 (0.02-0.15)** | 0.08 (0.02-0.14)** | 0.09 (0.02-0.15)** | 0.09 (0.03-0.16)** | 0.07 (0.02-0.13)* |
| Diff | 0.02 | 0.02 | 0.02 | 0.01 | 0.03 |
| % explained | 15.83 | 20.19 | 17.68 | 8.55 | 29.09 |
| **Other Asian** |  |  |  |  |  |
| Reduced | 0.03 (-0.04-0.09) | 0.03 (-0.04-0.1) | 0.03 (-0.04-0.09) | 0.03 (-0.04-0.09) | 0.03 (-0.04-0.1) |
| Full | 0.02 (-0.05-0.08) | 0.04 (-0.03-0.11) | 0.01 (-0.05-0.07) | 0.03 (-0.03-0.1) | 0.02 (-0.05-0.09) |
| Diff | 0.01 | -0.01 | 0.02 | 0.00 | 0.01 |
| % explained | 32.43 | -38.14 | 77.64 | -10.16 | 33.4 |
| **Other Ethnicity** |  |  |  |  |  |
| Reduced | 0.13 (0-0.26)* | 0.13 (0-0.25)* | 0.13 (0-0.25)* | 0.13 (0-0.26)+ | 0.12 (0-0.23)* |
| Full | 0.11 (-0.02-0.23)+ | 0.11 (-0.01-0.22)+ | 0.12 (-0.01-0.24)+ | 0.12 (-0.01-0.25)+ | 0.08 (-0.03-0.18) |
| Diff | 0.02 | 0.02 | 0.01 | 0.01 | 0.04 |
| % explained | 18.08 | 15.6 | 8.51 | 8.83 | 33.81 |
| **Other White** |  |  |  |  |  |
| Reduced | 0.07 (0.03-0.12)** | 0.07 (0.02-0.12)** | 0.07 (0.02-0.11)** | 0.07 (0.03-0.12)** | 0.07 (0.02-0.11)** |
| Full | 0.06 (0.02-0.11)** | 0.08 (0.03-0.13)*** | 0.06 (0.01-0.1)** | 0.07 (0.02-0.11)** | 0.06 (0.01-0.11)* |
| Diff | 0.01 | -0.01 | 0.01 | 0.00 | 0.01 |
| % explained | 9.97 | -14.6 | 12.43 | 4.25 | 9.92 |
| **Pakistani or Bangladeshi** |  |  |  |  |  |
| Reduced | 0.19 (0.12-0.26)*** | 0.19 (0.12-0.26)*** | 0.19 (0.12-0.26)*** | 0.19 (0.12-0.26)*** | 0.19 (0.12-0.26)*** |
| Full | 0.18 (0.1-0.25)*** | 0.11 (0.05-0.17)*** | 0.13 (0.06-0.2)*** | 0.18 (0.11-0.25)*** | 0.1 (0.03-0.17)** |
| Diff | 0.01 | 0.08 | 0.06 | 0.01 | 0.09 |
| % explained | 5.64 | 41.59 | 31.1 | 5.07 | 47.24 |

*** < 0.001, ** < 0.001, * <0.05, <0.10

1. Reduced model includes age, age squared and gender.

2. Demographic variables are: Country of origin, Partnership status, Presence of school age children, and Household containing person over 70.

3. Institutional variables are Education, Subjective financial situation, Tenure, Overcrowding, Area deprivation, and Access to car.

4. Community level variables are: Neighbourhood cohesion, Internal political efficacy, External political efficacy, Area racism, and Ethnic density.

5. Health variables are: Clinical vulnerability, Self-rated health, Limiting longstanding illness, GHQ-12, Life satisfaction, and Smoking

**Supplementary Table 4: Using Odds ratio from KHB regression models to compare ethnic inequalities before and after adjusting for variables in each domain.**

Coefficients presented are from a reduced model^1^, full model adjusting for variables in each domain, and difference between models,

| Ethnicity | Demographic^2^ | Institutional^3^ | Community4 | Health^%^ | All |
| --- | --- | --- | --- | --- | --- |
| (Reference White British) | OR (95% CI) | OR (95% CI) | OR (95% CI) | OR (95% CI) | OR (95% CI) |
| **Black** |  |  |  |  |  |
| Reduced | 9.33 (6.5-13.4)*** | 10.18 (6.97-14.88)*** | 9.79 (6.74-14.22)*** | 9.41 (6.51-13.6)*** | 10.75 (7.37-15.69)*** |
| Full | 7.69 (5.25-11.25)*** | 7.97 (5.42-11.71)*** | 7.35 (4.93-10.97)*** | 8.63 (5.97-12.48)*** | 6.62 (4.34-10.11)*** |
| Diff | 1.21 (1.00-1.47)* | 1.28 (0.96-1.71)+ | 1.33 (1.00-1.77)* | 1.09 (0.94-1.27) | 1.62 (1.03-2.56)* |
| **Indian** |  |  |  |  |  |
| Reduced | 2.43 (1.72-3.43)*** | 2.5 (1.74-3.57)*** | 2.45 (1.74-3.45)*** | 2.45 (1.74-3.45)*** | 2.56 (1.78-3.67)*** |
| Full | 2.29 (1.6-3.26)*** | 2.2 (1.53-3.18)*** | 1.86 (1.27-2.74)** | 2.47 (1.75-3.48)*** | 1.91 (1.25-2.93)** |
| Diff | 1.06 (0.88-1.29) | 1.13 (0.85-1.51) | 1.32 (0.98-1.76)+ | 0.99 (0.86-1.15) | 1.34 (0.84-2.12) |
| **Mixed** | |  |  |  |  |
| Reduced | 2.22 (1.47-3.34)*** | 2.27 (1.52-3.39)*** | 2.26 (1.5-3.4)*** | 2.22 (1.47-3.36)*** | 2.33 (1.55-3.49)*** |
| Full | 1.99 (1.32-3.00)*** | 1.96 (1.31-2.94)*** | 1.98 (1.3-3.01)*** | 2.09 (1.38-3.17)*** | 1.86 (1.22-2.84)** |
| Diff | 1.12 (0.96-1.30) | 1.16 (0.87-1.53) | 1.14 (0.88-1.47) | 1.06 (0.91-1.23) | 1.25 (0.82-1.9) |
| **Other Asian** |  |  |  |  |  |
| Reduced | 1.28 (0.75-2.21) | 1.31 (0.73-2.35) | 1.29 (0.74-2.25) | 1.30 (0.76-2.23) | 1.32 (0.74-2.35) |
| Full | 1.19 (0.67-2.11) | 1.44 (0.8-2.58) | 1.06 (0.6-1.88) | 1.33 (0.77-2.29) | 1.2 (0.64-2.25) |
| Diff | 1.08 (0.85-1.38) | 0.91 (0.69-1.21) | 1.22 (0.94-1.57) | 0.98 (0.84-1.13) | 1.1 (0.69-1.75) |
| **Other Ethnicity** |  |  |  |  |  |
| Reduced | 2.59 (1.21-5.53)* | 2.62 (1.23-5.6)* | 2.61 (1.22-5.58)* | 2.59 (1.19-5.63)* | 2.54 (1.23-5.27)* |
| Full | 2.24 (1.03-4.85)* | 2.30 (1.07-4.93)* | 2.42 (1.12-5.22)* | 2.42 (1.11-5.25)* | 1.92 (0.9-4.1)+ |
| Diff | 1.16 (0.95-1.42) | 1.14 (0.86-1.51) | 1.08 (0.84-1.4) | 1.07 (0.93-1.24) | 1.33 (0.85-2.07) |
| **Other White** |  |  |  |  |  |
| Reduced | 1.79 (1.3-2.47)*** | 1.81 (1.3-2.52)*** | 1.76 (1.27-2.44)*** | 1.79 (1.3-2.47)*** | 1.80 (1.28-2.52)*** |
| Full | 1.70 (1.19-2.41)** | 1.93 (1.38-2.7)*** | 1.64 (1.17-2.29)** | 1.75 (1.27-2.41)*** | 1.69 (1.16-2.47)** |
| Diff | 1.06 (0.85-1.31) | 0.94 (0.71-1.24) | 1.07 (0.84-1.36) | 1.02 (0.89-1.18) | 1.06 (0.68-1.66) |
| **Pakistani or Bangladeshi** | |  |  |  |  |
| Reduced | 3.57 (2.47-5.18)*** | 3.76 (2.61-5.44)*** | 3.7 (2.52-5.41)*** | 3.59 (2.5-5.16)*** | 3.88 (2.64-5.69)*** |
| Full | 3.36 (2.27-4.97)*** | 2.36 (1.61-3.47)*** | 2.62 (1.73-3.98)*** | 3.41 (2.37-4.91)*** | 2.23 (1.43-3.47)*** |
| Diff | 1.06 (0.89-1.27) | 1.59 (1.17-2.17)** | 1.41 (1.04-1.91)* | 1.05 (0.91-1.22) | 1.74 (1.09-2.79)* |

1. Reduced model includes age, age squared and gender.

2. Demographic variables are: Country of origin, Partnership status, Presence of school age children, and Household containing person over 70.

3. Institutional variables are Education, Subjective financial situation, Tenure, Overcrowding, Area deprivation, and Access to car.

4. Community level variables are: Neighbourhood cohesion, Internal political efficacy, External political efficacy, Area racism, and Ethnic density.

5.. Health variables are: Clinical vulnerability, Self-rated health, Limiting longstanding illness, GHQ-12, Life satisfaction, and Smoking

**Supplementary Table 5: Odds ratios for variables in the final model for COVID19 Vaccine Hesitancy**

|  | Odds Ratio | Confidence interval | | p |  |  | Odds Ratio | Confidence interval | | p |
| --- | --- | --- | --- | --- | --- | --- | --- | --- | --- | --- |
| *main exposure* |  |  |  |  |  | *Community level* |  |  |  |  |
| **Ethnicity** |  |  |  |  |  | **Neighbourhood cohesion** |  |  |  |  |
| White British | Ref |  |  |  |  | Per unit increase | 0.96 | 0.94 | 0.98 | 0.001 |
| Black | 6.62 | 4.34 | 10.11 | 0.000 |  | **Internal political efficacy** |  |  |  |  |
| Indian | 1.91 | 1.25 | 2.93 | 0.003 |  | Per unit increase | 0.95 | 0.91 | 0.98 | 0.006 |
| Mixed | 1.86 | 1.22 | 2.84 | 0.004 |  | **External political efficacy** |  |  |  |  |
| Other Asian | 1.20 | 0.64 | 2.25 | 0.561 |  | Per unit increase | 0.89 | 0.85 | 0.93 | 0.000 |
| Other Ethnicity | 1.92 | 0.90 | 4.10 | 0.093 |  | **Area racism** |  |  |  |  |
| Other white | 1.69 | 1.16 | 2.47 | 0.007 |  | Not at all | Ref |  |  |  |
| Pakistani and Bangladeshi | 2.23 | 1.43 | 3.47 | 0.000 |  | Not very | 0.81 | 0.69 | 0.96 | 0.012 |
|  |  |  |  |  |  | Fairly or very | 1.25 | 0.87 | 1.80 | 0.224 |
| *Demographic variables* |  |  |  |  |  | **Ethnic density** |  |  |  |  |
| **Age** |  |  |  |  |  | Proportion non-white (0-1) | 1.42 | 0.89 | 2.27 | 0.143 |
| Age in ten years | 1.41 | 1.02 | 1.97 | 0.040 |  |  |  |  |  |  |
| Age in ten years squared | 0.93 | 0.90 | 0.97 | 0.000 |  | *Institutional* |  |  |  |  |
| **Gender** |  |  |  |  |  | **Education** |  |  |  |  |
| Male | Ref |  |  |  |  | Degree | Ref |  |  |  |
| Female | 1.57 | 1.35 | 1.83 | 0.000 |  | A level | 1.39 | 1.11 | 1.76 | 0.005 |
| **Country of origin** |  |  |  |  |  | GCSE | 1.66 | 1.39 | 1.99 | 0.000 |
| Born in UK | Ref |  |  |  |  | None | 1.61 | 1.29 | 2.01 | 0.000 |
| Not born in UK | 1.14 | 0.87 | 1.48 | 0.340 |  | **Subjective finances** |  |  |  |  |
| **Partnership status** |  |  |  |  |  | Comfortably | Ref |  |  |  |
| Yes | Ref |  |  |  |  | Doing alright | 1.19 | 0.99 | 1.43 | 0.066 |
| No | 1.33 | 1.12 | 1.58 | 0.001 |  | Just about getting by | 1.65 | 1.30 | 2.10 | 0.000 |
| **School age children in home** |  |  |  |  |  | Difficult or Very difficult | 1.67 | 1.18 | 2.37 | 0.004 |
| None | Ref |  |  |  |  | **Housing Tenure** |  |  |  |  |
| One or more | 1.01 | 0.85 | 1.21 | 0.901 |  | Own outright | Ref |  |  |  |
| **Person over 70 in home** |  |  |  |  |  | Own with a mortgage | 0.89 | 0.74 | 1.08 | 0.253 |
| Zero | Ref |  |  |  |  | Socially rented | 1.20 | 0.90 | 1.61 | 0.214 |
| One or more | 0.68 | 0.50 | 0.94 | 0.020 |  | Private rented | 0.97 | 0.73 | 1.28 | 0.825 |
|  |  |  |  |  |  | Other | 1.16 | 0.31 | 4.39 | 0.824 |
| *Health variables* |  |  |  |  |  | **overcrowding** |  |  |  |  |
| **Clinical vulnerability** |  |  |  |  |  | Under-occupied | Ref |  |  |  |
| No Risk | Ref |  |  |  |  | Balanced | 0.76 | 0.61 | 0.95 | 0.015 |
| Moderate Risk | 0.76 | 0.64 | 0.91 | 0.002 |  | Overcrowded | 1.00 | 0.72 | 1.39 | 0.997 |
| High risk | 0.88 | 0.64 | 1.22 | 0.441 |  | **Area deprivation** |  |  |  |  |
| **Self-rated health** |  |  |  |  |  | Per decile increase | 0.95 | 0.92 | 0.98 | 0.000 |
| Excellent /Very good | Ref |  |  |  |  | **Access to car** |  |  |  |  |
| Good | 0.88 | 0.74 | 1.03 | 0.118 |  | At least once a day | Ref |  |  |  |
| Fair or poor | 0.88 | 0.67 | 1.14 | 0.324 |  | Most days | 0.75 | 0.61 | 0.92 | 0.006 |
| **Limiting long standing illness** |  |  |  |  |  | Once or twice a week | 0.79 | 0.66 | 0.95 | 0.012 |
| No | Ref |  |  |  |  | Less than that or never. | 0.84 | 0.68 | 1.03 | 0.101 |
| Yes | 0.94 | 0.79 | 1.11 | 0.449 |  |  |  |  |  |  |
| **loneliness** |  |  |  |  |  |  |  |  |  |  |
| Hardly ever or never | Ref |  |  |  |  |  |  |  |  |  |
| Some of the time | 0.83 | 0.70 | 0.98 | 0.028 |  |  |  |  |  |  |
| Often | 0.89 | 0.64 | 1.24 | 0.492 |  |  |  |  |  |  |
| **GHQ-12** |  |  |  |  |  |  |  |  |  |  |
| Per unit score | 0.99 | 0.98 | 1.01 | 0.418 |  |  |  |  |  |  |
| **Life satisfaction** |  |  |  |  |  |  |  |  |  |  |
| Per unit score | 1.04 | 0.99 | 1.10 | 0.102 |  |  |  |  |  |  |
| **Smoking** |  |  |  |  |  |  |  |  |  |  |
| No | Ref |  |  |  |  |  |  |  |  |  |
| Yes | 1.19 | 0.94 | 1.49 | 0.150 |  |  |  |  |  |  |
|  |  |  |  |  |  |  |  |  |  |  |

**Supplementary Table 6: Descriptive statistics for sample for which the individual racism measure was available (Note this was not just restricted to England)**

| Variable | N | % |
| --- | --- | --- |
|  |  |  |
| *Outcome* |  |  |
| **COVID19 Vaccine Hesitancy** |  |  |
| Not Hesitant | 766 | 64.81 |
| Hesitant | 416 | 35.19 |
|  |  |  |
| *Main exposures* |  |  |
| **Ethnicity** |  |  |
| Black | 182 | 9.68 |
| Indian | 269 | 14.31 |
| Mixed | 107 | 5.69 |
| Other Asian | 107 | 5.69 |
| Other Ethnicity | 37 | 1.97 |
| Other white | 266 | 14.15 |
| Pakistani and Bangladeshi | 214 | 11.38 |
|  |  |  |
| **Exposure to individual racism** |  |  |
| No exposure | 969 | 82.0 |
| 1 exposure | 104 | 8.8 |
| 2+ exposures | 109 | 9.2 |
| *Core demographic variables* |  |  |
|  |  |  |
| **Gender** |  |  |
| Male | 454 | 38.4 |
| Female | 728 | 61.6 |
|  |  |  |
|  | Mean | SD |
| **Age** | 48.1 | 14.5 |

**Supplementary Table 7: Odds ratios for vaccine hesitancy among minoritised ethnic people who participated in the wave 9 sample.**

|  |  | Odds ratio | Confidence intervals | | p value |
| --- | --- | --- | --- | --- | --- |
|  |  |  |  |  |  |
| **Exposure to racism** |  |  |  |  |  |
| 0 exposures |  | Reference |  |  |  |
| 1 exposure |  | 0.66 | 0.42 | 1.06 | 0.08 |
| 2+ exposures |  | 0.96 | 0.62 | 1.50 | 0.87 |
|  |  |  |  |  |  |
| **Ethnicity** |  |  |  |  |  |
| Indian |  | Reference |  |  |  |
| Black |  | 3.54 | 2.34 | 5.35 | 0.00 |
| Mixed |  | 0.86 | 0.52 | 1.42 | 0.55 |
| Other Asian |  | 0.48 | 0.27 | 0.84 | 0.01 |
| Other Ethnicity |  | 0.86 | 0.39 | 1.91 | 0.71 |
| Other white |  | 0.86 | 0.58 | 1.27 | 0.44 |
| Pakistani and Bangladeshi |  | 1.33 | 0.90 | 1.97 | 0.16 |
|  |  |  |  |  |  |
| **Age** |  | Reference |  |  |  |
| Age in ten years |  | 1.45 | 0.86 | 2.44 | 0.17 |
| Age in ten years squared |  | 0.93 | 0.88 | 0.98 | 0.01 |
|  |  |  |  |  |  |
| **Sex** |  |  |  |  |  |
| Male |  | Reference |  |  |  |
| Female |  | 1.43 | 1.10 | 1.87 | 0.01 |
|  |  |  |  |  |  |
